# Neurodevelopmental Outcomes of Very Low Birth Weight Infants With Transient Hypothyroxinaemia of Prematurity

**DOI:** 10.1111/apa.70302

**Published:** 2025-09-12

**Authors:** Sophie S. L. Yeow, Charis H. Y. Chung, Wilfred H. S. Wong, Khair Jalal, Mabel S. C. Wong, Wing‐Shan Queenie See

**Affiliations:** ^1^ Department of Paediatrics and Adolescent Medicine, Queen Mary Hospital The University of Hong Kong Hong Kong Hong Kong

**Keywords:** neurodevelopmental outcomes, prematurity, transient hypothyroxinaemia of prematurity, very low birth weight infants

## Abstract

**Aim:**

Transient hypothyroxinaemia of prematurity affects very low birth weight (VLBW) infants born prematurely. The relationship between transient hypothyroxinaemia of prematurity (THOP) and adverse neurodevelopmental outcomes is still controversial. The present study aimed to compare the neurodevelopmental outcomes between those with VLBW infants with THOP and those without.

**Methods:**

This was a single tertiary neonatal centre retrospective study comparing the neurodevelopmental outcome using Griffiths Mental Development Scales‐Extended Revised between VLBW infants (birth weight less than or equal to 1500 g) with THOP to those without based on one‐to‐one age and gender matching from 2011 to 2021.

**Result:**

Forty‐two VLBW infants with THOP and 42 VLBW infants without THOP were matched by age and gender. None of the infants with THOP received thyroxine supplementation. The prevalence of THOP is 6.86%. The mortality rate for infants with THOP was 14.3%. There was no significant difference in neonatal complications and neurodevelopmental outcomes between infants with THOP (70%) and infants without THOP (70.7%).

**Conclusion:**

We showed that there was no difference in neurodevelopmental outcomes between those with THOP without thyroxine supplementation and those without THOP.

**Abbreviations**
IVH: intraventricular haemorrhage
THOP: transient hypothyroxinaemia of prematurity
VLBW: very low birth weight

**Summary**

- The effect of transient hypothyroxinaemia of prematurity on neurodevelopmental outcomes and the benefit of thyroxine replacement is still unclear.
- The results from our centre show that there are no significant adverse neurodevelopmental outcomes for babies with THOP compared to those without THOP.
- More local studies are needed to investigate the local prevalence of THOP and the long‐term neurodevelopmental outcomes of those with THOP.

## 1 Introduction

Very low birth weight infants are defined as infants born with a birth weight lighter than or equal to 1500 g. Infants born prematurely are at risk of thyroid problems including, but not exhaustive, congenital hypothyroidism and transient hypothyroxinaemia of prematurity (THOP) [1]. THOP is defined as low thyroxine with normal or low TSH for gestational age [2]. It should be noted that normal reference ranges for thyroid function vary between different centres. THOP usually occurs within the first 1–2 weeks of life and usually resolves after 2–3 weeks [2]. The prevalence of THOP stated in literature is between 35% and 50% for preterm births [3, 4].

Currently there are no specific guidelines that recommend substitution or prophylactic treatment of THOP with thyroxine [5, 6]. Our centre does not routinely provide thyroxine supplementation to infants with THOP. Numerous studies have been done to investigate if there is any adverse effect of THOP on neurodevelopmental outcome, but results have been conflicting [6]. It is widely known that for preterm infants there is a delayed rise in TSH for congenital hypothyroidism. Therefore, in our centre, we monitor the thyroid function of preterm infants regularly. Once on Day 10–14 of life, then the 4th week of life, and then every 4 weeks until reaching term and achieving 2 kg. If the thyroid function test was abnormal, the thyroid function test may be repeated earlier depending on the severity of the abnormality. This study aims to investigate THOP in our locality and compare the neurodevelopmental outcomes of VLBW infants with THOP without thyroxine supplementation to those without THOP.

## 2 Methods

This was a single tertiary neonatal centre retrospective study. The inclusion criteria include very low birth weight infants (birth weight less than or equal to 1500 g) born or transferred to our hospital during the neonatal period from 2011 to 2021. Data was collected from the Hospital Authority Clinical Management System and the Vermont Oxford Neonatal Network after our research study was approved by the Hospital Authority Clinical Research Ethics Review Committee. Exclusion criteria included infants who had passed before discharge or had congenital hypothyroidism (both transient and permanent). Infants fulfilling criteria for VLBW would have their thyroid function test reviewed. THOP is defined as low thyroxine with normal or low TSH for gestational age and sex, according to our laboratory reference [7, 8, 9, 10] (Tables 1 and 2), that normalises after a few weeks without treatment. VLBW infants with normal thyroid function were matched one‐to‐one with each THOP case based on gender and gestation. After matching, the controls were randomly sorted. The first one from the sorted list will be selected. If there are no case matches by the exact gestation, controls will be chosen with a gestation ±1 week. Data on sex, gestational age, mode of delivery, maternal chorioamnionitis, Apgar score, birthweight, neonatal complications and neurodevelopmental outcomes were collected. We use the Vermont Oxford Network definition of BPD, which is the need for oxygen requirement at 36 weeks postmenstrual age in a preterm neonate with compatible radiographic findings.

**TABLE 1:** Plasma TSH reference interval.

Sex	Age	Plasma TSH (mIU/L)
Female	0 year–< 6 days	0.7–15.2
	6 days–< 2 months	0.66–6.04
	2 months–< 1 year	0.44–5.44
	1 year–< 7 year	0.70–5.97
	7 year–< 12 year	0.60–4.84
	12 year–999 year	0.27–4.20
Male	0 year–< 6 days	0.70–15.2
	6 days–< 2 months	0.72–6.57
	2 months–< 1 year	0.44–4.86
	1 year–< 7 year	0.70–5.97
	7 year–< 12 year	0.60–4.84
	12 year–999 year	0.27–4.20

**TABLE 2:** Plasma free T4 for gestation reference interval.

Gestation (week)	pmol/L
≤ 5	11.6–23.0
> 5–6	11.9–23.8
> 6–7	12.1–24.5
> 7–9	12.1–24.5
> 9–10	11.9–23.9
> 10–11	11.7–23.3
> 11–12	11.4–22.8
> 12–13	11.2–22.2
> 13–14	11.0–21.6
> 14–15	10.7–21.0
> 15–16	10.5–20.5
> 16–20	10.1–19.4
> 20–32	9.2–17.4
> 32–36	9.0–17.0
> 36	9.2–17.4

All VLBW infants would have routine developmental assessment at 18 to 22 months corrected age. The developmental assessment in our centre is Griffiths Mental Development Scales‐Extended Revised. Developmental delay was defined as having a general quotient in one domain of 78 or less. Global developmental delay was defined as having delay in more than one developmental domain. Developmental domains include Locomotor, Personal‐Social, Hearing and Speech, Eye and Hand, and Performance. General quotient is the average score of all the developmental domains combined.

Statistical analyses were done using the statistical package for the social sciences version 24. The continuous variables are expressed as means and standard deviation. Comparisons were done using a paired *t*‐test and McNemar test. Univariate analysis was done for individual factors. Significant difference was defined as a *p*‐value < 0.05.

## 3 Results

There were a total of 714 VLBW infants born or transferred to our tertiary unit from 2011 to 2021. Sixty‐six of these infants passed away before discharge (seven had THOP). One infant passed away after discharge. In the end, there were 42 VLBW infants who fulfilled the diagnostic criteria for THOP. None of the infants with THOP received thyroxine supplementation, and they all recovered spontaneously. We had 42 control cases without THOP. The prevalence of THOP according to our data is 6.8%. The mortality rate for babies with THOP was 14.3%. The mortality rate for infants without THOP was 8.9. Causes of death for infants with THOP include extreme prematurity, septicaemia, and necrotizing enterocolitis. The lowest free thyroxine level in our THOP group was 5.8 pmol/L.

Statistical analysis showed there was no significant difference in demographic data between the THOP and control groups, which include the birth weight, mode of delivery, antenatal steroids, dopamine infusion, maternal chorioamnionitis and Apgar score (Table 3).

**TABLE 3:** Demographics.

	Overall	THOP	Control group	
	(*N* = 84)	(*N* = 42)	(*N* = 42)	
	*N* (%)	*N* (%)	*N* (%)	
	Mean ± SD	Mean ± SD	Mean ± SD	*p*
	Median (Q3, Q1)	Median (Q3, Q1)	Median (Q3, Q1)	Paired *t*‐test/(McNemar test)
Gender				
Male	52 (61.9%)	26 (61.9%)	26 (61.9%)	(1.000)
Female	32 (38.1%)	16 (38.1%)	16 (38.1%)	
Gestational age at birth (weeks)	26.8 ± 2.4	26.6 ± 2.3	27.1 ± 2.4	0.076
Birth weight (gram)	890.8 ± 270.8	887.5 ± 285.6	894.1 ± 258.5	0.859
Mode of delivery				
Normal spontaneous delivery and others	46 (53.5%)	23 (53.5%)	23 (54.8%)	(1.000)
Caesarean section related	39 (45.3%)	20 (51.3%)	19 (45.2%)	
Antenatal steroids				
Yes	79 (94.0%)	39 (95.1%)	40 (95.2%)	(1.000)
No	4 (4.8%)	2 (4.9%)	2 (4.8%)	
Dopamine infusion				
Yes	28 (33.7%)	14 (34.1%)	14 (33.3%)	(1.000)
No	55 (66.3%)	27 (65.9%)	28 (66.7%)	
Maternal chorioamnionitis				
Yes	41 (49.4%)	17 (41.5%)	24 (57.1%)	(0.210)
No	42 (50.6%)	24 (58.5%)	18 (42.9%)	
Apgar score, 1 min	5 (7, 3)	5 (7, 3)	5 (7, 3)	0.886
Apgar score, 5 min	8 (9, 7)	8 (9, 7)	8 (9, 6.75)	0.760

For neonatal complications, there was no significant difference between the THOP and control groups for bronchopulmonary dysplasia (71.4% in THOP group and 69% in control group, *p* = 0.727), intraventricular haemorrhage (95.2% in THOP group and 81% in control group, *p* = 0.109), small‐for‐gestational age (4.8% in THOP group and 9.8% in control group, *p* = 0.625), necrotizing enterocolitis (26.2% in THOP group and 14.3% in control group, *p* = 0.267), sepsis (28.6% in THOP group and 16.7% in control group, *p* = 0.227), periventricular leukomalacia (2.4% in THOP group and 9.5% in control group, *p* = 0.375) and retinopathy of prematurity (63.4% in THOP group and 59.5% in control group, *p* = 1) (Table 4). However, there was a significant increase in the risk of patent ductus arteriosus in the THOP group compared to the control group (71.4% in THOP group and 52.4% in control group, *p* = 0.035) (Table 4).

**TABLE 4:** Neonatal complications.

Variables	Overall	THOP	Control group	
	(*N* = 84)	(*N* = 42)	(*N* = 42)	
	*N* (%)	*N* (%)	*N* (%)	*p*
	Mean ± SD	Mean ± SD	Mean ± SD	McNemar test
Small‐for‐gestational age				
Yes	6 (7.2%)	2 (4.8%)	4 (9.8%)	0.625
No	77 (92.8%)	40 (95.2%)	37 (90.2%)	
Dopamine				
Yes	28 (33.7%)	14 (34.1%)	14 (33.3%)	1.000
No	55 (66.3%)	27 (65.9%)	28 (66.7%)	
Bronchopulmonary dysplasia				
Yes	59 (70.2%)	30 (71.4%)	29 (69.0%)	0.727
No	25 (29.8%)	12 (28.6%)	13 (31.0%)	
Patent ductus arteriosus				
Yes	52 (61.9%)	30 (71.4%)	22 (52.4%)	0.035
No	32 (38.1%)	12 (28.6%)	20 (47.6%)	
Intraventricular haemorrhage				
Yes	74 (88.1%)	40 (95.2%)	34 (81.0%)	0.109
No	10 (11.9%)	2 (2.4%)	8 (19.0%)	
Necrotizing enterocolitis				
Yes	17 (20.2%)	11 (26.2%)	6 (14.3%)	0.267
No	67 (79.8%)	31 (73.8%)	36 (85.7%)	
Sepsis				
Yes	19 (22.6%)	12 (28.6%)	7 (16.7%)	0.227
No	65 (77.4%)	30 (71.4%)	35 (83.3%)	
Periventricular leukomalacia				
Yes	5 (6.0%)	1 (2.4%)	4 (9.5%)	0.375
No	79 (94.0%)	41 (97.6%)	38 (90.5%)	
Retinopathy of prematurity				
Yes	51 (61.4%)	26 (63.4%)	25 (59.5%)	1.000
No	32 (38.6%)	15 (36.6%)	17 (40.5%)	

There was no significant difference in neurodevelopmental outcomes between the THOP and control groups (95.2 ± 9.8 in THOP group, 91.4 ± 17.3 in control group, *p* = 0.292) (Table 5). Nine patients with THOP did not have developmental assessments performed. There were 23 infants out of 33 infants in the THOP group who had developmental delay in at least one domain (70%). There were 10 infants with normal development (30%). Sixteen patients with THOP had delay in only one developmental domain (48.5%). Six patients with THOP had global developmental delay (18.2%). For the control group, there were 29 patients who had delay in at least one developmental domain (70.7%). Only one patient in the control group did not perform a developmental assessment. Twenty‐one infants had developmental delay in only one domain (51%). Eight patients in the control group had global developmental delay (19.5%). Twelve infants in the control group had normal development (29%). Language delay was the most affected developmental domain, followed by gross motor delay in both the THOP and control groups. For the control group, 25 infants had language delay (61%) and 5 infants had gross motor delay (12%). For the THOP group, 20 infants had language delay (60%) and 3 infants had gross motor delay (9%).

**TABLE 5:** Neurodevelopmental outcomes.

Variables	Overall	THOP		*p*	Control group		*p*
	(*N* = 84)	(*N* = 42)		Independent‐samples	(*N* = 42)		Independent‐samples
	Mean ± SD	Mean ± SD		*t*‐test	Mean ± SD		*t*‐test
		No/Mild IVH (Grade 1–2)	Severe IVH (Grade 3–4)		No/Mild IVH (Grade 1–2)	Severe IVH (Grade 3–4)	
General quotient	94.4 ± 13.9	96.0 ± 9.3	92.6 ± 14.4	0.521	95.7 ± 12.0	72.7 ± 34.6	0.006
Locomotor	101.1 ± 18.2	103.3 ± 12.0	89.0 ± 22.7	0.057	103.6 ± 16.2	75.8 ± 41.6	0.010
Personal‐social	100.2 ± 17.2	102.9 ± 13.4	108.5 ± 19.9	0.464	99.7 ± 16.0	76.5 ± 32.7	0.018
Hearing and speech	77.1 ± 21.0	76.3 ± 22.4	86.0 ± 22.1	0.424	78.5 ± 20.0	61.0 ± 18.0	0.102
Eye and hand	93.0 ± 14.8	93.1 ± 11.4	90.3 ± 3.8	0.627	95.3 ± 12.9	73.8 ± 39.3	0.018
Performance	101.0 ± 18.4	104.6 ± 13.6	88.5 ± 15.9	0.038	102.3 ± 15.9	76.0 ± 44.4	0.015

An independent *t*‐test was done to analyse the effect of the severity of intraventricular haemorrhage (IVH) on the neurodevelopmental outcomes. A further analysis was done to compare if this effect differs between THOP and the control group. Patients with IVH were divided into two groups based on severity. One group consisted of patients with no IVH, Grade 1, or 2 IVH. The second group consisted of patients with severe IVH, which includes Grade 3 or 4 IVH. The result of the independent *t*‐test demonstrated that there were significant differences in General Quotient, Locomotor, Personal‐Social, Eye and Hand coordination, and Performance between the No/Mild grade IVH and Severe grade IVH group in the control group. The mean (SD) value of the General Quotient in the Severe IVH group (72.7 ± 34.6) is significantly lower than the mean (SD) of the No/Mild grade IVH group (95.7 ± 12.0), with a *p*‐value of 0.006. The mean (SD) value of Locomotor in the Severe IVH group (75.8 ± 41.6) is significantly lower than the mean (SD) of the No/Mild IVH group (103.6 ± 16.2) with a *p*‐value of 0.01. The mean (SD) value of Personal‐Social in the Severe IVH group (76.5 ± 32.7) is significantly lower than the mean (SD) of the No/Mild group (99.7 ± 16.0) with a *p*‐value of 0.018. The mean (SD) value of Eye and Hand coordination in the Severe IVH group (73.8 ± 39.3) is significantly lower than the mean (SD) of the No/Mild grade IVH group (95.3 ± 12.9) with a *p*‐value of 0.018. The mean (SD) value of Performance in the Severe IVH group (76.0 ± 44.4) is significantly lower than the mean (SD) of the No/Mild grade IVH group (102.3 ± 15.9) with a *p*‐value of 0.015. All domains except Hearing and Speech show *p*‐values < 0.05, indicating statistically significant differences.

In contrast, the THOP group showed less pronounced differences, with only the Performance domain reaching significance. The mean (SD) value of Performance in the Severe IVH group was 88.5 ± 15.9, which is significantly lower than the mean (SD) of the No/Mild grade IVH group of 104.6 ± 13.6, with a *p*‐value of 0.038. G*Power version 3.1.9 was employed to determine the study's sample size power. A post hoc analysis involved 84 participants, consisting of 42 controls and 42 cases. This configuration enables the identification of an effect size with an odds ratio exceeding 3.6, ensuring a statistical power of 80% for a two‐tailed McNemar test. Regarding the continuous variable assessed using a paired *t*‐test, the sample size of 84 can detect a minimum difference with an effect size of 0.31 (Cohen's D), achieving a statistical power of 80% at a 5% significance level with two tails.

## 4 Discussion

The relationship between THOP and neurodevelopmental outcomes has been studied for the past few decades. A recent review of the literature on THOP and its impact on neurodevelopmental outcomes was published by An Eerdekens et al. in 2018 [6]. There were two multi‐centre, double blind randomised placebo‐controlled trials included in this review. Both trials did not show any benefit of thyroxine supplementation on neurodevelopmental outcomes. The rest were observational longitudinal studies. Of particular note was the two observational longitudinal cohort studies performed by Hollanders et al., which had a larger number of subjects and were published within the last 10 years. The one published in 2015 showed no association between THOP and neurodevelopmental outcomes at the age of 19 years old [11]. The other one published in 2016 showed that THOP was associated with more behavioural problems at the age of 19 years old [12]. The majority of the studies in the review showed THOP is related to worse neurodevelopmental outcomes; however, they were all observational case–control studies done more than 10 years ago. The review concluded that thyroxine supplementation for those with THOP may be beneficial, but more studies are needed [6].

Transient hypothyroxinaemia of prematurity can be explained physiologically. The growing fetus is completely reliant on maternal thyroid hormone up until mid‐gest ation [13]. After mid‐gestation, the hypothalamic–pituitary axis matures and the foetus starts to produce its own thyroid hormone [13]. In term infants, there is a surge of TSH at birth, which results in elevated thyroxine levels in the first 24 h of life. In preterm babies, however, thyroxine levels are low in the first week of life. This can be explained by the immature hypothalamic–pituitary axis, decreased thyroxine binding globulin by the liver, immature thyroid gland hormone production, and effects of non‐thyroid illness syndrome [13]. Thyroid hormone is vital to brain development; even a short period of deficiency during periods of brain development could have consequences, which has been demonstrated in animal and clinical studies. However, results from clinical trials have been conflicting about the benefits of thyroxine supplementation for THOP on neurodevelopmental outcomes.

The prevalence of THOP in our study was 5.88%, which is much lower than the quoted prevalence in literature of 30% to 50%. This can be explained by a few reasons. Firstly, we have a higher proportion of transient hypothyroidism compared to other studies (23% compared to 2% in the USA) [14]. This may be due to subclinical iodine deficiency present in up to 35.8% of pregnant women as reported in a local pilot study [15]. VLBW infants, in turn, are more susceptible in view of other post‐natal stressors and a developing hypothalamic–pituitary axis. A local study showed that the incidence of permanent congenital hypothyroidism in VLBW infants was almost five times higher than reported in the New England Newborn Screening base from January 1989 to June 2002 [16]. As many infants with laboratory evidence of congenital hypothyroidism, whether transient or permanent, were excluded, this left a small remaining group of infants that fulfilled the laboratory criteria of THOP. Secondly, thyroid function was only checked once for each baby within the first 2 weeks of life. Therefore, there is a chance that THOP was underdiagnosed in our study. This is the first study done investigating the prevalence of THOP in Hong Kong. Further local studies on the prevalence of THOP and transient congenital hypothyroidism in VLBW infants may help us to understand better. Our study had several limitations. It was a retrospective study, with some incomplete data. Infants with no developmental assessment done was not included in the study. It was also a single centre study.

We studied the effect of the severity of IVH on neurodevelopmental outcomes, and whether this effect was different between THOP and the control group. We found that when compared to the control group with severe IVH, the THOP group with severe IVH was associated with less severe neurodevelopmental outcomes. This has not been reported in other studies, and we do not draw any conclusion from this association. Further studies are needed to evaluate this association before a conclusion can be made.

Furthermore, even though we tried our best to adjust for important confounding factors, there are other confounding factors that would have influenced the neurodevelopmental outcomes such as socioeconomic status. In conclusion, our results showed that there is no significant difference in neurodevelopmental outcomes between VLBW infants with THOP and those without. Further studies on local data are needed to verify this and to study the longer‐term effects of THOP on neurodevelopmental outcomes.

## 5 Conclusion

Our study showed that infants with THOP without thyroxine supplementation did not have any significant adverse neurodevelopmental outcomes compared to infants without THOP.

## Author Contributions

**Sophie S. L. Yeow:** conceptualisation, data curation, formal analysis, writing‐original draft. **Charis H. Y. Chung:** data curation, formal analysis. **Wilfred H. S. Wong:** formal analysis, methodology. **Khair Jalal:** supervision, writing – review and editing. **Mabel S. C. Wong:** supervision, writing – review and editing. **Wing‐Shan Queenie See:** conceptualisation, methodology, supervision, writing – review and editing.

## Conflicts of Interest

The authors declare no conflicts of interest.

## Data Availability

The data that supports the findings of this study are available in the Supporting Information of this article.

## References

[1] D. A. Fisher , “Thyroid System Immaturities in Very Low Birth Weight Premature Infants,” Seminars in Perinatology 32, no. 6 (2008): 387–397. 19007675 10.1053/j.semperi.2008.09.003

[2] F. Williams and R. Hume , “The Measurement, Definition, Aetiology and Clinical Consequences of Neonatal Transient Hypothyroxinemia,” Annals of Clinical Biochemistry 48 (2011): 7–22. 20930033 10.1258/acb.2010.010174

[3] S. Uhrmann , K. H. Marks , M. J. Maisels , H. E. Kulin , M. Kaplan , and R. Utiger , “Frequency of Transient Hypothyroxinaemia in Low Birthweight Infants. Potential Pitfall for Neonatal Screening Programmes,” Archives of Disease in Childhood 56 (1981): 214–217. 7212760 10.1136/adc.56.3.214 PMC1627144

[4] J. H. Anthony , D. A. Lyal , H. K. Alan , and A. F. Delbert , “Significance of Transient Postnatal Hypothyroxinemia in Premature Infants With and Without Respiratory Distress Syndrome,” Pediatrics 68 (1981): 494–498. 6798539

[5] D. A. Osborn and R. W. Hunt , “Prophylactic Postnatal Thyroid Hormones for Prevention of Morbidity and Mortality in Preterm Infants,” Cochrane Database of Systematic Reviews 2007 (2007): CD005948. 17253571 10.1002/14651858.CD005948.pub2 PMC9004229

[6] A. Eerdekens , L. Langouche , G. Van den Berghe , J. Verhaeghe , G. Naulaers , and C. Vanhole , “Review Shows That Thyroid Hormone Substitution Could Benefit Transient Hypothyroxinaemia of Prematurity but Treatment Strategies Need to Be Clarified,” Acta Paediatrica 108, no. 5 (2019): 792–805, 10.1111/apa.14685. 30537292

[7] Cobas , “Reference Intervals for Children and Adults,” Elecsys Thyroid Tests 2008: 15 and Roche RRs for Adults and Children, 2008:67. Elecsys RR Study.

[8] “Central 95 Percentile of QEH Outpatient Data (2013.07.01 to 2014.12.31),” Excluded Abnormal FT4 & TSH (TSH <0.10 & >10.0 mIU/L).

[9] Roche , “Cobas e 801 TSH Package Insert,” Ref 07028091190, 2016–03, V1.0 (2.5th–97.5th percentile).

[10] L. Y. Yuen , M. H. M. Chan , D. S. Sahota , et al., “Development of Gestational Age‐Specific Thyroid Function Test Reference Intervals in Four Analytic Platforms Through Multilevel Modeling,” Thyroid 30, no. 4 (2020): 598–608. 31910112 10.1089/thy.2019.0323

[11] J. J. Hollanders , J. Israels , S. M. van der Pal , P. H. Verkerk , J. Rotteveel , and M. J. Finken , “No Association Between Transient Hypothyroxinemia of Prematurity and Neurodevelopmental Outcome in Young Adulthood,” Journal of Clinical Endocrinology and Metabolism 100 (2015): 4648–4653. 26480285 10.1210/jc.2015-3078

[12] J. J. Hollanders , S. M. van der Pal , P. H. Verkerk , J. Rotteveel , and M. J. Finken , “Transient Hypothyroxinemia of Prematurity and Problem Behavior in Young Adulthood,” Psychoneuroendocrinology 72 (2016): 40–46. 27343725 10.1016/j.psyneuen.2016.06.008

[13] S. H. LaFranchi , “Thyroid Function in Preterm/Low Birth Weight Infants: Impact on Diagnosis and Management of Thyroid Dysfunction,” Frontiers in Endocrinology 12 (2021): 666207. 34211436 10.3389/fendo.2021.666207 PMC8239410

[14] R. S. Brown , R. L. Bellisario , D. Botero , et al., “Incidence of Transient Congenital Hypothyroidism due to Maternal Thyrotropin Receptor‐Blocking Antibodies in Over One Million Babies,” Journal of Clinical Endocrinology and Metabolism 81 (1996): 1147–1151. 8772590 10.1210/jcem.81.3.8772590

[15] A. W. Kung , T. T. Lao , L. C. Low , R. W. Pang , and J. D. Robinson , “Iodine Insufficiency and Neonatal Hyperthyrotropinaemia in Hong Kong,” Clinical Endocrinology 46 (1997): 315–319. 9156041 10.1046/j.1365-2265.1997.1310960.x

[16] Y. Y. Chee , K. Y. Wong , and L. Low , “Review of Primary Hypothyroidism in Very Low Birthweight Infants in a Perinatal Centre in Hong Kong,” Journal of Paediatrics and Child Health 47, no. 11 (2011): 824–831. 21435074 10.1111/j.1440-1754.2011.02033.x

